# *Enterobacter cloacae* Periprosthetic Joint Infection After Bariatric Surgical Anastomotic Leak

**DOI:** 10.5435/JAAOSGlobal-D-19-00095

**Published:** 2020-01-09

**Authors:** Justin T. DesLaurier, Joyce J. Chung, Awais K. Hussain, Michael J. Patetta, Mark H. Gonzalez, Menachem M. Meller

**Affiliations:** From the University of Illinois College of Medicine (Mr. DesLaurier, Ms. Chung, Dr. Hussain, Dr. Patetta, Dr. Gonzalez), Chicago, Illinois, and the Mercy Health System (Dr. Meller), Philadelphia, Pennsylvania.

## Abstract

Four weeks after a bilateral total knee arthroplasty (TKA), an immunocompetent, 61-year-old, Caucasian man presented with a periprosthetic joint infection (PJI) of the left knee by *Enterobacter cloacae* (an enteric bacteria). The most likely source of his infection was due to an anastomotic leak after a bariatric surgery done 6 months before TKA. There is a growing focus on stratifying the risk of PJI after TKA. Hematogenous seeding of enteric bacteria leading to PJI is an unexplored risk that will become more prevalent as bariatric procedures before TKA continue to increase in frequency. We present a patient who demonstrates this PJI risk with a rare microbe (*E cloacae*).

The incidence of class III obesity, defined as a body mass index greater than 40 kg/m^2^, continues to increase in the United States.^1^ In addition, these individuals represent a higher proportion of those undergoing prosthetic joint arthroplasty.^2^ Previous studies have identified the increased risks in doing total knee arthroplasties (TKAs) in individuals with increased body mass indexes (BMIs).^3^ Consequently, these affected individuals have increased rates of being declined TKAs and are referred to for weight loss and possible bariatric surgery.^4^ The AAOS guidelines do not currently recommend a specific time interval between a gastric bypass and subsequent TKA. In addition, these guidelines have highlighted the evaluation of outcomes for patients with gastric bypass surgery before TKA as an area of future research.^5^ Current trends in bariatric surgery consist of endoscopic lap bands, sleeves, and bypass (Roux-en-Y) procedures, listed in order of increasing severity regarding their physiologic effect. Surgical complications categorized as “early” (occurring within two weeks of the procedure) included anastomotic leak, gastrointestinal bleeding, intestinal obstruction, and incorrect Roux limb reconstruction.^6^ Surgical complications categorized as “late” (occurring after 2 weeks) included anastomotic strictures, marginal ulceration, and gastrogastric strictures.^7^ None of the described complications included orthopaedic manifestations. Anastomotic leaks after gastric bypass procedures are rare (1%); most require reintervention and the majority recover from this event. Risk factors of anastomotic leak include open surgery, revision surgery, and the use of an abdominal drain.^8^

We present a case of periprosthetic joint infection (PJI) in an individual who underwent bilateral TKA 6 months after successful weight loss from an endoscopic sleeve surgery. The metabolic parameter goals including improvement of the patient's metabolic profile and reduction of BMI were realized. The postoperative TKA course was initially normal, and there was routine perioperative management. Four weeks after his TKA, the patient presented to the emergency department with positive SIRS criteria and mental status changes. He had variable and vague abdominal reports. A sepsis workup revealed an anastomotic leak at the sleeve bypass site. Further infectious workup revealed markedly elevated serum C-reactive protein (CRP) and ESR, as well as elevated bilateral joint aspirate white blood cell (WBC) counts. His left knee culture was positive for *Enterobacter cloacae*. A DAIR (débridement, antibiotics, and implant retention) procedure and a course of antibiotics resolved the right knee, whereas the left required a 2-stage revision after a failed DAIR procedure. Long-term postoperative complications followed. The patient was informed that the data concerning this case would be submitted for publication, and the patient provided his consent.

## Case Report

A 61-year-old Caucasian man presented with a history of insulin-dependent diabetes, hypertension, coronary artery disease (stent placed), post-traumatic stress disorder, morbid obesity (BMI 46.1 kg/m^2^), and severe osteoarthritis of both knees. In September 2017, he underwent a sleeve gastrectomy in an effort to reduce weight and improve his overall health and metabolic profile. Over a 6-month period, his weight improved dramatically to a BMI of 31.9 and his blood glucose improved from ∼300 mg/dL back to normal. However, his knee symptoms failed to improve despite the use of a cane and NSAIDs. The patient received intra-articular injections in the distant past, but not in proximity to his TKA.

In March 2018, the patient underwent a bilateral TKA and had a normal course. He was transferred to an acute care rehabilitation center for one week and was subsequently discharged home with the use of a rolling walker. His incisions were dry and intact.

In April 2018, the patient presented to the emergency department with fever, change in mental status, poor nutritional intake, and purulent drainage from the left knee incision. He was found to be tachycardic and tachypneic with an elevated lactate level and respiratory alkalosis. Radiographs obtained from both knees revealed well-fixated implants. The patient's serum WBC count was within normal limits; however, his inflammatory markers were elevated, with an ESR of 45 mm/hr and a CRP of 65.8. Noncontrast CT of the abdomen identified a localized anastomotic leak complicating his previous bariatric surgery. A joint fluid analysis revealed 48,831 WBCs in the right knee and 32,725 in the left knee. Joint fluid culture results yielded *E cloacae* in the left knee.

**Figure 1:**
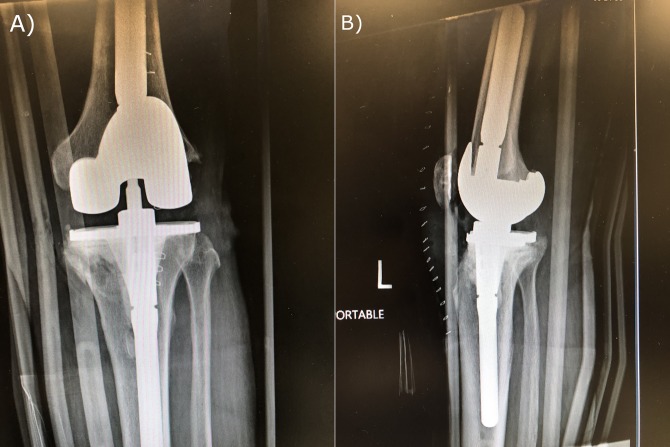
Radiograph showing postoperative AP (**A**) and lateral (**B**) views of the left knee after a total knee arthroplasty revision taken 1 day after surgery (November 2, 2018).

**Figure 2:**
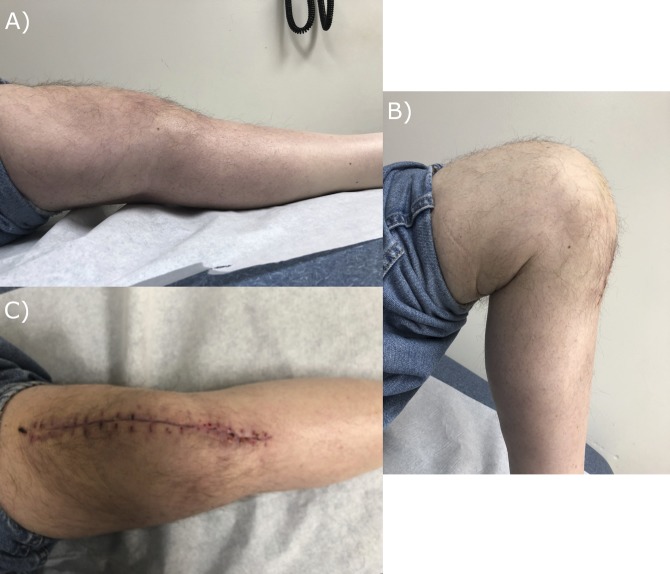
Clinical photographs which demonstrate postrevision left knee extension (**A**), flexion (**B**), and alignment (**C**) taken 4 weeks after surgery (November 27, 2018).

The patient was then brought to the operating room where he underwent a same-day DAIR procedure, followed by culture-specific parenteral antibiotics. His postoperative course was complicated by the acute onset of renal failure attributed in part to his aminoglycoside antibiotic. The antibiotic was then changed to cefepime. Parenteral antibiotics were continued for 6 weeks, followed by a 2-week hiatus and reaspiration. The right knee was deemed sterile, but the left knee tested positive for persistent *Enterobacter* infection and failed a subsequent débridement and retention effort. At the time of his readmission, his BMI remained stable at 31 kg/m^2^. He was submitted to a 2-stage revision with a cephalosporin-impregnated cement spacer and subsequent implant. Left knee explant and antibiotic spacer placement occurred in August 2018. The patient underwent his final revision left TKA in November 2018 (Figure 1). As of a 4-week follow-up, the patient had a stable, comfortable knee with good knee function (Figure 2).

The patient returned to the hospital in March 2019 with bilateral knee pain and swelling as well as drainage of the left knee. He was found to have bacteremia positive for *Peptostreptococcus* and *Propionibacterium* and PJIs of *Peptostreptococcus* (left knee) and *Staphylococcus epidermidis* (right knee). In addition, he presented with acute kidney injury requiring hospitalization and hemodialysis for 1 month before discharge. Infections were managed with a 6-week IV course of daptomycin and ampicillin-sulbactam. The patient returned to the hospital 2 weeks after discharge with bleeding from the left knee incision site and anemia and managed with plasma infusion and OR débridement. One week later (late April), he returned with altered mental status and increased bilateral knee pain shortly after cessation of his antibiotic course. He had not followed up with infectious disease staff as directed by that time. Blood and urine studies showed hypoglycemia, inflammatory markers indicative of infection, and progression of chronic kidney disease. Infectious disease suspected inflammatory flare-ups because of chronic bilateral PJIs and noted that the patient may require lifelong antibiotic management. He may additionally require amputation in the future if the infections cannot be managed conservatively.

## Discussion

As TKA becomes more readily available and done in increasing numbers, it is important to stratify risks and costs of specific subgroups. Management of risks, particularly in the bariatric population, will continue to be important for this procedure. Data from the Healthcare Cost and Utilization Project state that 752,941 knee arthroplasties were done in 2014, which was approximately 40% more procedures than in 2005.^9^ The number of TKA procedures annually is expected to increase 5-fold from 2005 to 2030,^10^ further indicating the need for the cost stratification of TKAs. The economic burden of a single TKA revision surgery has been estimated at $49,360 per procedure, which totaled $2.7 billion in hospital charges in 2012. This number is expected to exceed $13 billion by 2030 by one study.^11^ Because these numbers continue to grow each year, every risk factor for TKA revision surgery will have the potential to have a profound effect on healthcare costs.

Certain risk factors for TKA revision surgeries occur commonly and have been extensively studied. These include cigarette smoking,^12^ poorly controlled diabetes,^13,14^ hypertension,^15^ chronic kidney disease,^16^ and other contributors to an elevated Charlson score. These risk factors regularly circulate in the literature as the leading comorbidities with poor TKA outcomes. These outcomes often lead to revision surgeries, other adverse patient events, and resource consumption. Owing to the prevalence and extensive research into such risk factors, preoperative optimizations have been developed. Such optimizations include completing dental care and eliminating caries,^17,18^ undergoing cardiac stents and improving preoperative cardiac care,^19^ and optimizing diabetes and renal function,^20,21^ all of which have been demonstrated to be effective in decreasing adverse outcomes.

Although progress has been made on preoperative optimization of TKAs, many emerging risk factors for adverse outcomes are yet to be addressed within standard TKA guidelines, likely because of their uncommon or subtle nature. It is important for clinicians to keep this in mind when assessing risk in patients with unconventional histories.

We present a case report describing a sequence of adverse outcomes despite adherence by the patient's healthcare team to the standard guidelines. This sequence was primarily characterized by a post-TKA hematogenous spread of enteric bacteria to the knee secondary to an anastomotic leak. The culture was specific for *E cloacae*, which is part of the normal gut microbiome. It is a gram-negative, rod-shaped facultative anaerobe sensitive to gentamicin and cefepime. We should emphasize that the anastomotic leak was discovered coincidentally as part of a sepsis workup with only vague abdominal reports. We present this case as an example where hematogenous seeding of a TKA could be a danger for patients with a history of previous bariatric procedure or other types of bowel anastomosis, especially in the case of an anastomotic leak. Although this case is a catastrophic example of both short-term and long-term complications resulting from this unanticipated outcome, we believe it emphasizes the potential impact of this unexplored risk. Further studies are needed to quantify this risk and develop best practice in risk stratification and optimization in such patients.
